# Imaging soliton dynamics in optical microcavities

**DOI:** 10.1038/s41467-018-06031-5

**Published:** 2018-09-03

**Authors:** Xu Yi, Qi-Fan Yang, Ki Youl Yang, Kerry Vahala

**Affiliations:** 0000000107068890 grid.20861.3d T.J. Watson Laboratory of Applied Physics, California Institute of Technology, Pasadena, CA 91125 USA

## Abstract

Solitons are self-sustained wavepackets that occur in many physical systems. Their recent demonstration in optical microresonators has provided a new platform for the study of nonlinear optical physics with practical implications for miniaturization of time standards, spectroscopy tools, and frequency metrology systems. However, despite its importance to the understanding of soliton physics, as well as development of new applications, imaging the rich dynamical behavior of solitons in microcavities has not been possible. These phenomena require a difficult combination of high-temporal-resolution and long-record-length in order to capture the evolving trajectories of closely spaced microcavity solitons. Here, an imaging method is demonstrated that visualizes soliton motion with sub-picosecond resolution over arbitrary time spans. A wide range of complex soliton transient behavior are characterized in the temporal or spectral domain, including soliton formation, collisions, spectral breathing, and soliton decay. This method can serve as a visualization tool for developing new soliton applications and understanding complex soliton physics in microcavities.

## Abstract (web summary)

In order to study the dynamics of solitons in microresonators, which underlie nonlinear phenomena like Kerr comb generation, both high temporal resolution and long record times are needed. Here, the authors develop a coherent sampling method to directly image the temporal behavior of solitons.

## Introduction

Temporal solitons are indispensable in optical fiber systems^1^ and exhibit remarkable nonlinear phenomena^2^. The potential application of solitons to buffers and memories^3,4^, as well as interest in soliton physics has stimulated approaches for experimental visualization of multi-soliton trajectories. Along these lines, the display of soliton trajectories in a co-moving frame^5^ allows an observer to move with the solitons and is being used to monitor soliton control and interactions of all types in fiber systems^5–12^. However, this useful data visualization method relies upon soliton pulse measurements that are either limited in bandwidth (pulse resolution) or record length. It is therefore challenging to temporally resolve solitons over the periods often required to observe their complete evolution. For example, the time-lens method^13^ can provide the required femtosecond-resolution, but has a limited record length set by the pump pulse. Also, while the relative position of closely spaced soliton complexes^11^ can be inferred over time from their composite dispersive Fourier transform (DFT) spectra^14^, Fourier inversion requires the constituent solitons to have similar waveforms which restricts the generality of the technique. Efforts that combine these two methods were also reported very recently^15,16^.

These limitations are placed in sharp focus by recent demonstrations of soliton generation in microcavities^17–23^. This new type of dissipative soliton^24^ was long considered a theoretical possibility^3^ and was first observed in optical fiber resonators^4^. Their microcavity embodiment poses severe challenges for imaging of dynamical phenomena by conventional methods, because multi-soliton states feature inherently closely spaced solitons. Preliminary real-time measurements using time lens^25^, and direct detection^26^ have been explored, but were limited in either recording length or pulse resolution. Nonetheless, the compactness of microcavity-based soliton systems has practical importance for miniaturization of frequency comb technology^27^ through chip-based microcombs^28,29^. Indeed, spectroscopy systems^30,31^, coherent communication^32^, ranging^33,34^, and frequency synthesis^35^ demonstrations using the new miniature platform have already been reported. Moreover, the unique physics of the new soliton microcavity system has led to observation of many unforeseen physical phenomena involving compound soliton states, such as Stokes solitons^36^, soliton number switching^37^ and soliton crystals^38^.

In this work, we report imaging of a wide range of soliton phenomena in microcavities. Soliton formation, collisions^10^, breathing^6,39–41^, Raman shifting^42,43^, as well as soliton decay are observed. Significantly, femtosecond-time-scale resolution over arbitrary time spans (distances) is demonstrated (and required) in these measurements. Also, real-time spectrograms are produced along-side high-resolution soliton trajectories. These features are derived by adapting coherent linear optical sampling^44^ and electric-field cross-correlation^45^ to the problem of microcavity soliton imaging. Beyond the necessity to employ a new method for imaging soliton motion in microcavities, the high-repetition rate of microcavity solitons (tens of gigahertz and higher) is advantageous in sampling-based recording of motion.

## Results

### Coherent sampling of soliton motion

**Fig. 1:**
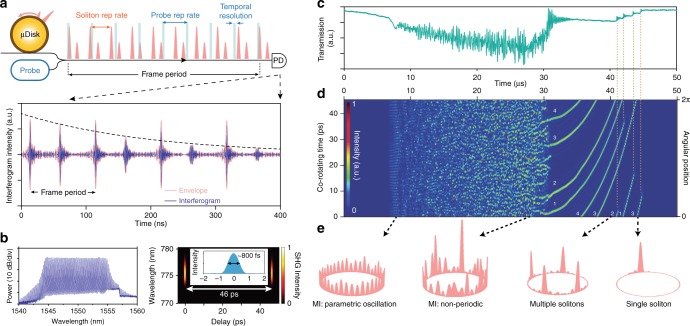
Coherent sampling of dissipative Kerr soliton dynamics. **a** Conceptual schematic showing microcavity signal (red) combined with the probe sampling pulse train (blue) using a bidirectional coupler. The probe pulse train repetition rate is offset slightly from the microcavity signal. It temporally samples the signal upon photo detection to produce an interferogram signal shown in the lower panel. The measured interferogram shows several frame periods during which two solitons appear with one of the solitons experiencing decay. PD: photodetector. **b** Left panel is the optical spectrum and right panel is the second harmonic generation (SHG) intensity of the probe electro-optic comb measured with FROG (pulse repetition period is shown as 46 ps). An intensity autocorrelation in the inset shows a full-width-at-half-maximum pulse width of 800 fs. **c** Microresonator pump power transmission when the pump laser frequency scans from higher to lower frequency. Multiple steps indicate the formation of solitons. **d** Imaging of soliton formation corresponding to the scan in **c**. The *x*-axis is time and the *y*-axis is time in a frame that rotates with the solitons (full scale is one round-trip time). The right vertical axis is scaled in radians around the microcavity. Four soliton trajectories are labeled and fold-back into the cavity coordinate system. The color bar gives their signal intensity. **e** Soliton intensity patterns measured at four moments in time are projected onto the microcavity coordinate frame. The patterns correspond to initial parametric oscillation^51^ in the modulation instability (MI) regime^3, 4^, non-periodic behavior (MI regime), four soliton and single soliton states^4, 17^

To image the soliton trajectories, a separate optical probe pulse stream is generated at a pulse rate that is close to the rate of the solitons to be imaged in the microcavity. The small difference in these rates causes a pulse-to-pulse temporal shift of the probe pulses relative to the microcavity signal pulses as illustrated in Fig. 1a. By heterodyne detection of the combined streams, the probe pulses coherently sample the microcavity signal producing a temporal interferogram^46,47^ shown in Fig. 1a. Ultimately, the time shift per pulse accumulates so that the sampling repeats in the interferogram at the frame rate which is described below, and is close in value to the difference of sampling and signal rates. Probe pulses have a sub-picosecond temporal resolution that enables precise monitoring of the temporal location of the soliton pulses. Moreover, the coherent mixing of probe and soliton pulses allows extraction of each soliton’s spectral evolution by fast Fourier transform of the interferogram. In principle, the probe pulses can be generated by a second microcavity soliton source operating in steady state. However, in the present measurement, an electro-optic (EO) comb is used^45,48,49^. The EO comb pulse rate is conveniently adjusted electronically to match the rates of various phenomena being probed within the microcavity.

The soliton signal is produced by a 3 mm diameter silica wedge resonator with a free-spectral-range (FSR) of 22 GHz and intrinsic quality factor above 200 million^18,50^. The device generates femtosecond soliton pulses when pumped at frequencies slightly lower than a cavity resonant frequency^18^. To sample the 22 GHz soliton signal the EO comb was formed by modulation of a tunable continuous-wave (CW) laser. The EO comb features ~1.3 THz optical bandwidth (within 1 dB power variation) and an 800 fs full-width-at-half-maximum (FWHM) pulse width is measured by frequency-resolved optical gating (FROG) and autocorrelation as shown in Fig. 1b. Further details on the experimental setup are provided in the Methods section. In all presented measurements, the pump laser of the resonator scans linearly from higher to lower frequency to initiate parametric oscillation^51^ in the microcavity followed by chaotic dynamics. Ultimately, step-like features are observable in the resonator transmitted power (Fig. 1c) indicating the formation of soliton states^17^. The typical pump power and laser scan speed are ~70 mW and ~1 MHz/μs, respectively.

### Measuring multiple soliton trajectories

As described above, heterodyne detection of the soliton signal and the EO-comb pulse produces the electrical interferogram. The period of the signals in the interogram is adjusted by tuning the EO-comb repetition rate. In the initial measurements, it is set to ~10 MHz lower than the rate of the microcavity signal so that the nominal period in the interferogram is ~100 ns. To display the interferogram signal a co-rotating frame is applied. First, a frame period *T* is chosen that is close to the period of signals of interest in the interferogram. Integer steps (i.e., *mT*) are plotted along the *x*-axis while the interferogram is plotted along the *y*-axis, but offset in time by the *x*-axis time step (i.e., *t-mT*). To make connection to the physical time scale of the solitons, the *y*-axis time scale is compressed by the same bandwidth compression factor (*T*×FSR) that accompanies the sampling process (see Discussion). The *y*-axis scale is accordingly set to span one microcavity round-trip time. A typical measurement plotted in this manner is given in Fig. 1d. Because this way of plotting the data creates a co-rotating reference frame, a hypothetical soliton pulse with an interferogram period equal to the frame rate *T* would appear as a horizontal line in Fig. 1d. On the other hand, slower (higher) rate solitons would appear as lines tilted upward (downward) in the plot. In creating the imaging plot, a Hilbert transformation is applied to the interferogram followed by taking the square of its amplitude to produce a pulse envelope intensity profile. The vertical co-rotating time axis can be readily mapped into the soliton angular position within the circular microcavity as shown in Fig. 1d (right vertical axis).

Imaging of soliton formation and multi-soliton trajectories is observable in Fig. 1d. For comparison with the transmitted power, the time-axis scale is identical in Fig. 1c, d. As the pump laser frequency initially scans towards the microcavity resonant frequency its coupled power increases. At ~8 μs the resonator enters the modulation instability regime^3,4,17^. Initially, a periodic temporal pattern is observable in Fig. 1d corresponding to parametric oscillation^51^. Soon after, the cavity enters a regime of non-periodic oscillation. At ~31 μs, this regime suddenly transitions into four soliton pulses. The soliton positions evolve with scan time and disappear one-by-one. All solitons have upward curved trajectories, showing that the soliton repetition rate decreases as the scan progresses. This soliton rate shift is caused by the combination of the Raman self-frequency shift effect and anomalous dispersion in the silica resonator^42,52^ and a similar effect on soliton trajectory is observed in optical fiber resonators^43^. Supplementary Movie 1 provides the corresponding multi-soliton motion around the microcavity. Finally, the cavity states at four moments in time are plotted within the circular microcavity in Fig. 1e. These correspond to parametric oscillation, non-periodic modulational instability, four soliton, and single soliton states.

### Observation of soliton collisions

**Fig. 2:**
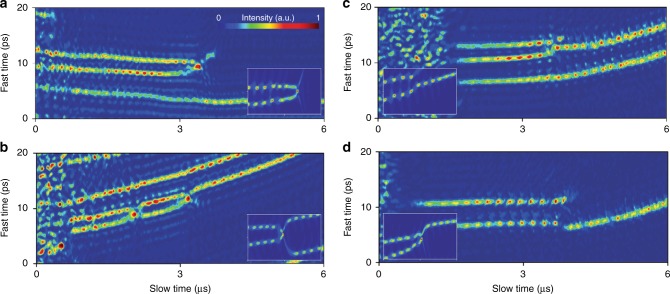
Measurements of non-repetitive soliton events. **a** Two solitons collide and annihilate. A wave splash appears in the collision. **b** Two solitons survive a collision, but collide again and one soliton is annihilated. **c** Two solitons collide and merge into a single soliton. **d** A soliton hops in location when another soliton is annihilated. The measurement frame rate is 50 MHz in all panels. Inset panels show similar collision events from numerical simulation, including the appearance of the wave splash (inset in **a**)

A variety of non-repetitive multi and single soliton phenomena were measured in both temporal and spectral domains. To enable more rapid imaging the repetition rate of EO comb was adjusted to produce an interferogram at a rate of approximately 50 MHz. The frame period, *T*, was then reduced accordingly to ~20 ns. Figure 2 presents observations of two solitons interacting. Soliton annihilation is observed in Fig. 2a, wherein two solitons move toward each other, collide, create an intense peak upon collision and then disappear. A new phenomena, a wave splash, is observed immediately following the collision. Though not discussed, this feature appears in recently reported simulations^12^. In Fig. 2b, two solitons collide but quickly recover and then collide again, after which point one soliton is annihilated. Figure 2c shows a third example in which solitons merge and a single soliton emerges. In a fourth case shown in Fig. 2d, soliton hopping accompanies annihilation of a soliton. Interestingly, all soliton collisions are observed at the beginning of soliton formation (in the soliton breathing regime). After this regime, the soliton relative motion quickly stabilizes preventing collisions. This stabilization process is investigated in a later section. Also, as noted earlier, the observation of these complex motions requires measurement of events in close temporal proximity over long time spans. Finally, numerical simulations of soliton collisions are shown as inset panels in Fig. 2. The collisional features observed in experiments, including the wave splash in Fig. 2a, are reproduced in the simulations.

### Breather soliton spectrograms

**Fig. 3:**
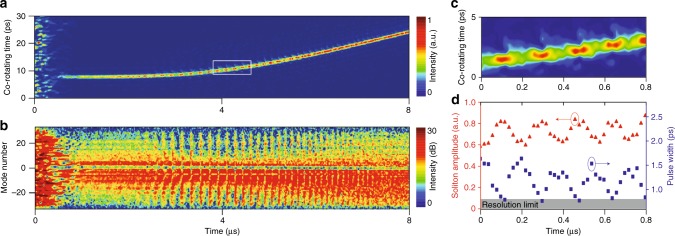
Temporal and spectral measurements of breather solitons. **a** Motion of a single soliton state showing peak power breathing along its trajectory. **b** Spectral dynamics corresponding to **a**. The *y*-axis is the relative longitudinal mode number corresponding to specific spectral lines of the soliton. Mode zero is the pumped microcavity mode. The soliton spectral width breaths as the soliton peak power modulates. The spectrum is widest when peak power is maximum. **c** Zoom-in view of the white rectangular region in **a**. **d** Soliton amplitude and pulse width breathing corresponding to **c**. The frame rate is 50 MHz for all panels

Figure 3 shows measurement of a breathing soliton^39^ in both the temporal and frequency domains. The intensity of an individual breather soliton is imaged in Fig. 3a. Spectral breathing was explored in fiber-ring resonators using the DFT method^6^. In the current work, the spectral breathing is observed by applying a Fourier transform to the interferogram signal^47^. Figure. 3b shows the resulting spectrogram plotted over the same time interval as Fig. 3a wherein the spectrum is widest when the breather soliton has its maximum peak power. This spectrum also reveals the changing breather period with frequency scan, which has previously been observed by measurement of soliton power^26,41^. A zoom-in of the soliton temporal breathing is shown in Fig. 3c. The combined high frame rate and sub-ps temporal resolution enable the corresponding amplitude and pulse width of the breather to be extracted and these are plotted in Fig. 3d. As an observation unrelated to the breathing action, the soliton spectral envelope in Fig. 3b is continuously red shifted in frequency by the Raman self-frequency shift^18,42^ as its average power increases (increasing time in the plot).

### Tracking relative soliton motion

**Fig. 4:**
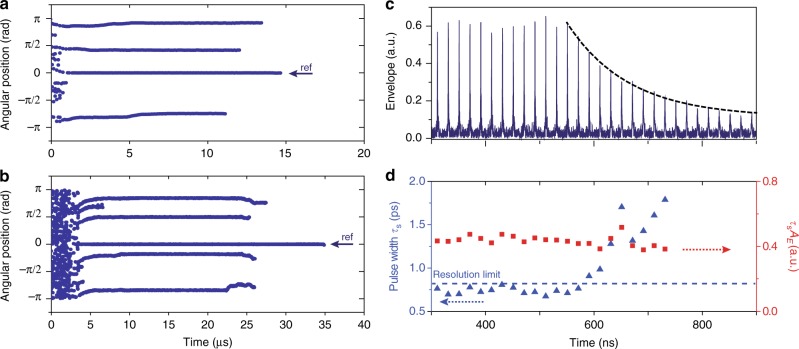
Measurement of relative soliton positions and soliton decay. **a** Plot of the relative positions of four solitons while the pumping laser frequency is scanned (high to low). The reference soliton, used to establish zero angular position, is indicated and all solitons have stable relative positions after only several μs of motion. **b** The relative positions of five solitons is measured versus time as the pump laser frequency is scanned. The soliton relative positions stabilize and then destabilize at 22 μs. The frame rates for **a** and **b** are 10 and 50 MHz, respectively. **c** Interferogram envelope showing a single soliton experiencing decay. An exponential fitting is given as the dashed black line. **d** The measured pulse width (blue) is plotted versus time and its resolution limit (dashed blue line) is set by the EO comb pulse width. The product of soliton amplitude and pulse width is plotted in red

Monitoring relative soliton position in real time is important for study of soliton optical memories^3,4^, their interaction and control^5,9^ as well as in soliton crystals^38^. Previously, microcavity soliton relative positions have been measured by autocorrelation^53^, frequency-resolved optical gating^17^ and synchronized cross-correlation^38^. However, with an update rate limited by a mechanical delay line, these methods are only useful for measurement of steady-state phenomena. In this work, relative soliton positions can be measured in real time from the interferogram thereby enabling study of their relative motion dynamics. To plot soliton relative position, one soliton is selected to be the reference (i.e., zero point of the angular position) and the angular position relative to the reference soliton is defined from −*π* to *π*. Two representative measurements are shown in Fig. 4a, b wherein the laser frequency is scanned from high to low frequency. Even though the reference soliton round-trip rate is changing as the laser frequency is scanned (see, for example, Fig. 1d), the solitons experience extended stable motion relative to one another. In Fig. 4a, the solitons stabilize relative to each other within a few microseconds after formation and in Fig. 4b, the relative positions are stable from 9 to 22 μs and then destabilize. It is believed that the stabilization of solitons is related to the presence of a dispersive wave caused by an avoided mode crossing^54^. Note that ultimately, all of the solitons in both panels are annihilated when the laser is tuned beyond the existence detuning range^17^.

### Characterization of soliton decay

Finally, soliton decay is analyzed using the sampling method. The measurement results are shown in Fig. 4c, d. In the experiment, the pump laser frequency is continuously tuned toward lower frequencies. After soliton formation, at some point the cavity-laser frequency detuning exceeds the soliton existence range and the soliton decays^17,18^. Figure 4c shows the interferogram signal just before and during the decay. Pulse widths (*τ*_s_) are extracted during the decay process and are plotted in Fig. 4d. Also plotted in Fig. 4d is the product of pulse width and soliton peak amplitude (*A*_*E*_). Curiously, the soliton pulse width and peak amplitude preserve the same soliton product relationship as prior to decay. This is an indication that the decaying soliton pulse in the microcavity is constantly adapting itself to maintain the soliton waveform. A similar behavior is known to occur for conventional solitons in optical fiber^55^. To the authors knowledge, this is the first time this behavior has been observed in real time. In the Methods section the amplitude decay of the soliton in the interferogram trace is analyzed to extract a decay time and the cavity *Q* factor.

### Importance of high soliton repetition rate

Coherent sampling induces a large bandwidth compression of the ultrafast signal that is equal to the sampling rate divided by the difference in the signal rate and the sampling rate. This compression is well known in the related techniques of dual-comb spectroscopy^47^ and dual-comb ranging^46^, and is also present in sampling of optical signals by four-wave mixing in optical fibers^56^. In order to avoid spectral folding, the compressed signal bandwidth must lie within half of the EO comb sampling rate^46,47^ (the Nyquist condition for sampling). As shown in the Methods section, this basic condition establishes the following relationship between temporal resolution (*τ*), frame rate (*f*) and the sampling rate (approximately the microcavity free-spectral-range, FSR): *f*<*τ*FSR^2^/2. This condition also reveals the quadratic importance of high sampling rates (equivalently large FSRs and correspondingly large soliton repetition rates) to create fast frame rates. In the current system, a temporal resolution of <1 ps combined with a 22 GHz sampling rate can enable frame rates as high as 200 MHz.

### Numerical simulation of multi-soliton trajectories

**Fig. 5:**
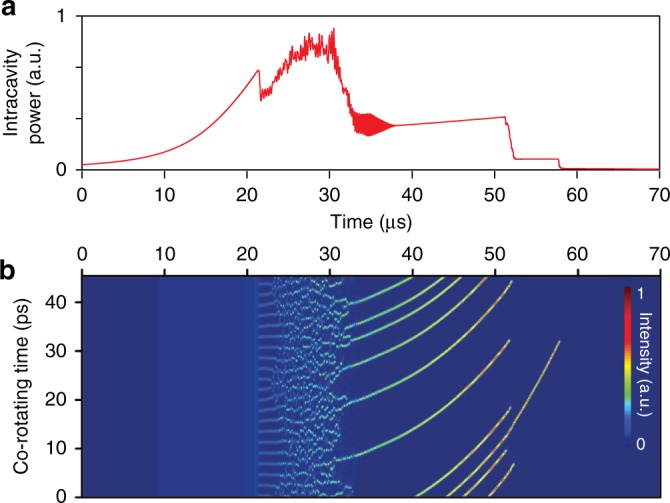
Simulation of multiple microcavity soliton formation and their trajectories. **a** Simulated intracavity power plotted versus time as the pumping laser is tuned across a cavity resonance from higher to lower frequencies. The step features correspond to the formation of solitons. **b** Simulation results corresponding to panel **a** and showing the formation of multiple solitons. In the simulation, the Raman effect and avoided mode crossing are included

Soliton dynamics are governed by the Lugiato-Lefever (LL) equation^57^ augmented by Raman^42,52^ and avoided mode crossing^58^ effects. The LL equation can be simulated numerically using the split-step method^55^. Simulated intracavity power versus temporal profiles for soliton formation are presented Fig. 5a and, for comparison with the imaging data in Fig. 1d, the corresponding simulated multi-soliton trajectories are plotted in Fig. 5b. In the simulation, the laser frequency is linearly scanned from higher to lower frequency. Moreover, the Raman effect and one avoided mode crossing are included in the simulation. Concerning the vertical axis scale, it is noted that because the periodicity of the soliton interferogram signals varies by <1 % during the scan, the vertical co-rotating time axis can be readily mapped into the soliton angular position axis within the circular microcavity as shown in Fig. 1d, e. The features of soliton formation and evolution observed in Fig. 1d compare well with the numerical simulation.

## Discussion

Imaging of nonlinear dynamical phenomena including complex soliton interactions with high temporal/spatial resolution over arbitrary time/length spans has been demonstrated. The temporal resolution in the current experiment is limited to 800 fs, however, resolution at the tens of fs level is possible by spectrally broadening the EO comb^59^ used for coherent sampling. It is also possible to replace the EO-comb with a microcomb that is closely matched to the FSR of a microcavity to be sampled. Such matching has been used in dual-microcomb spectroscopy demonstrations^30,31^. In this case, even higher sampling rates would be possible that would enable gigahertz-scale frame rates. Moreover, an implementation of soliton sampling produced within a single microresonator comb has also been recently reported^60^. The coherent sampling method can serve as a general real-time state visualization tool to monitor the dynamics of microcavity systems. It would provide an ideal way to monitor the formation and evolution of soliton complexes such as Stokes solitons^36^, soliton number switching^37^, and soliton crystals^38^. It can also be used to monitor the state of chip-based optical memories based on microresonator solitons.

## Methods

### Detailed experimental setup

**Fig. 6:**
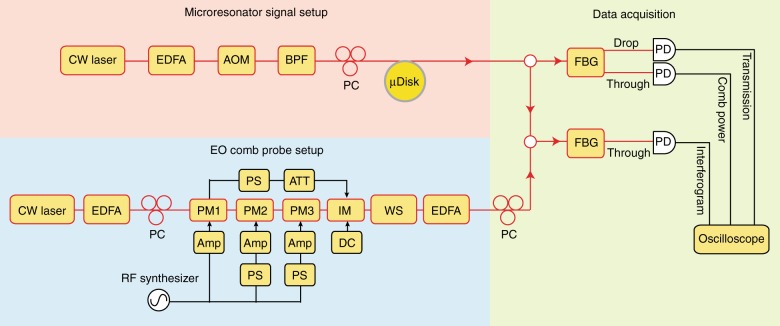
Experimental setup. Schematic showing the three functional sections in the experiment. CW laser: continuous-wave laser; EDFA: erbium-doped-fiber-amplifier; AOM: acousto-optic modulator; BPF: bandpass filter; PC: polarization controller; PM: phase modulator; IM: intensity modulator; PS: phase shifter; ATT: attenuator; Amp: RF amplifier; DC: voltage source; WS: optical waveshaper; FBG: fiber-Bragg-gating; PD: photodetector

Figure 6 divides the experimental setup into three sections. In the microresonator section, a tunable, continuous-wave (cw) laser is used to pump the microcavity for production of solitons. An erbium-doped fiber amplifier (EDFA) amplifies its power to 500 mW and an acousto-optic modulator (AOM) is used for rapid control of power to the microcavity. A tunable bandpass filter (BPF) is used to block the spontaneous emission noise from the EDFA. The pump is coupled into the microcavity through a tapered-fiber^61,62^. The emitted power from the microcavity (along with transmitted pump power) is split by a 90/10 fiber coupler. Ten percent of the power is sent to a fiber-Bragg grating (FBG) filter to separate the pump power and the microcomb power. The drop port output is the pump power transmission, while the through-port output is the comb power. Both the pump transmission and the microcomb power are detected with photodetectors (125 MHz bandwidth). The other 90% of the power is combined with the electro-optic (EO) modulation comb sampling pulse using a second fiber coupler.

In the EO comb setup, a pump laser is amplified by an EDFA to 200 mW and then phase modulated by three tandem lithium niobate modulators. The EO comb and microcavity setup can share the same pump laser when the acousto-optic modulator can provide a frequency offset higher than half of the electrical bandwidth of the interferogram signal (to avoid frequency folding). This is the case in Fig. 1. However, they can also use separate pump lasers, which is demonstrated in Figs. 2 and 3. In this case, the frequency sweep range of the soliton pump laser is always less than the frame rate to ensure that the soliton spectrum can be accurately inferred from Fourier transform of the interferogram. The modulators are driven by amplified electrical signals (frequency close to 22 GHz) that are synchronized by electrical phase shifters. The output power of the electrical amplifiers is 33 dBm. The phase modulated pump is then coupled to an intensity modulator to select only portions of the waveform with a uniform chirp. The intensity modulator is driven by the recycled microwave signal from the external termination port of the first phase modulator. The phase and amplitude of the modulation are controlled by electrical attenuators and phase shifters. A programmable line-by-line waveshaper is used to flatten the EO comb optical spectrum and to nullify the linear chirping so as to form a transform-limited sinc-shaped temporal pulse. The average power from the waveshaper output is around 100 μW. The EO pulses are amplified by an EDFA before combining with the microresonator signal.

In the interferogram measurement, the microcavity signal and the EO pulses are combined in a 90/10 coupler and are then detected by a fast photodetector with 50 GHz bandwidth. An FBG filter is used to block the pump laser of the microcavity to avoid saturation in the photodetector. All photodetected signals are recorded using a 4 GHz bandwidth, 20 GSa/s sampling rate oscilloscope. The center frequencies (compression factors) for the interferograms are around 0.7 GHz (2200) and 2.1 GHz (440) for the 10 MHz and 50 MHz frame rates, respectively.

### Time constant in soliton decay

In the soliton decay process, the average intracavity energy decays exponentially and its time constant equals the dissipation rate of the cavity (*κ* = *ω/Q*), where *ω* is the optical frequency and *Q* is the loaded cavity *Q* factor. For large cavity-laser frequency detuning^17,52^, the average intracavity energy is approximately the soliton energy, $\tau _sA_E^2$, such that

1 $$\tau _s(t)A_E^2\left( t \right) = \tau _s\left( 0 \right)A_E^2(0)e^{ - \kappa t}.$$

When the dissipation rate is relatively small compared to soliton Kerr nonlinear shift, the dissipation is a perturbation and the pulse maintains its soliton waveform^55^. The corresponding balance of dispersion and Kerr-nonlinearity requires that the product of soliton amplitude and pulse width be constant. This condition was also verified experimentally in Fig. 4d^52,55^,

2 $$\tau _s(t)A_E(t) = \tau _s(0)A_E(0).$$

Inserting eq. (2) into eq. (1) gives,

3 $$A_E(t) = A_E(0)e^{ - \kappa t},\tau _s(t) = \tau _s(0)e^{\kappa t},A_E^2(t) = A_E^2(0)e^{ - 2\kappa t}.$$

In particular, the soliton amplitude decays at the cavity dissipation rate, the pulse width exponentially grows, and the soliton peak power decays twice as fast as the cavity dissipation rate. In the experiment, the fitted decay constant of the soliton amplitude is 133 ns, which corresponds to *κ/*(2*π*) = 1.2 MHz giving *Q* = 161 million. This value is in reasonable agreement with the measured loaded-*Q* factor of 140 million.

### Nyquist condition for sampling

In the EO comb sampling process the optical to electrical conversion is accompanied by a large bandwidth compression of the sampled signal. In effect, sampling stretches the time scale so that, for example, the optical temporal resolution (*τ*) is stretched to *τ*×FSR*/f* after conversion to the electrical signal where *f* is the frame rate given by *f* ≈ FSR – *f*_comb_. This stretching means that the THz EO comb resolution bandwidth is compressed to an electrical bandwidth of *f*/(*τ*FSR). To avoid non-sensical signals in the electrical spectrum, the compressed bandwidth should lie within the Nyquist frequency set by the FSR^46^. This gives the condition *f*/(*τ*FSR)<FSR/2, or *f*<*τ*FSR^2^/2. In practice, when the oscilloscope bandwidth (*f*_osc_) is smaller than the Nyquist frequency, the interferogram signal will be limited by the oscilloscope instead of the Nyquist frequency, such that *f/*(*τ*FSR)<*f*_osc_, or *f<τf*_osc_FSR. This is, in fact, the case in the present measurement as the oscilloscope bandwidth is 4 GHz while the Nyquist frequency is 11 GHz. In addition, the frequency components of the interferogram signal must be positive to avoid frequency folding near zero frequency. This requires that the carrier frequency of the interferogram signal is larger than half of the electrical bandwidth. In the present measurement, the carrier frequency is the frequency offset between the EO comb pump laser and the microcavity pump laser (defined as ΔΩ). As a result, this condition is expressed as ΔΩ>*f*/(2*τ*FSR).

## Electronic supplementary material

Description of Additional Supplementary Files

Supplementary Movie 1

## Data Availability

The data that support the plots within this paper and other findings of this study are available from the corresponding author upon reasonable request.

## References

[1] Kivshar, Y. S. & Agrawal, G. *Optical solitons: from fibers to photonic crystals* (Academic press, 2003).

[2] Dudley JM, Dias F, Erkintalo M, Genty G (2014). Instabilities, breathers and rogue waves in optics. Nat. Photon..

[3] Wabnitz S (1993). Suppression of interactions in a phase-locked soliton optical memory. Opt. Lett..

[4] Leo F (2010). Temporal cavity solitons in one-dimensional Kerr media as bits in an all-optical buffer. Nat. Photon..

[5] Jang JK, Erkintalo M, Murdoch SG, Coen S (2013). Ultraweak long-range interactions of solitons observed over astronomical distances. Nat. Photon..

[6] Luo, K., Jang, J. K., Erkintalo, M., Murdoch, S. G. & Coen, S. Real-time spectral evolution of breathing temporal cavity solitons. In *European Quantum Electronics Conference*, EF_P_18 (Optical Society of America, 2015).

[7] Luo K, Jang JK, Coen S, Murdoch SG, Erkintalo M (2015). Spontaneous creation and annihilation of temporal cavity solitons in a coherently driven passive fiber resonator. Opt. Lett..

[8] Anderson M, Leo F, Coen S, Erkintalo M, Murdoch SG (2016). Observations of spatiotemporal instabilities of temporal cavity solitons. Optica.

[9] Jang, J. K., Erkintalo, M., Coen, S. & Murdoch, S. G. Temporal tweezing of light through the trapping and manipulation of temporal cavity solitons. *Nat. Commun*. **6**, 7370 (2015). 10.1038/ncomms8370 26104146

[10] Jang JK (2016). Controlled merging and annihilation of localised dissipative structures in an AC-driven damped nonlinear schrödinger system. New J. Phys..

[11] Herink G, Kurtz F, Jalali B, Solli D, Ropers C (2017). Real-time spectral interferometry probes the internal dynamics of femtosecond soliton molecules. Science.

[12] Anderson M (2017). Coexistence of multiple nonlinear states in a tristable passive Kerr resonator. Phys. Rev. X.

[13] Foster MA (2008). Silicon-chip-based ultrafast optical oscilloscope. Nature.

[14] Goda K, Jalali B (2013). Dispersive fourier transformation for fast continuous single-shot measurements. Nat. Photon..

[15] Ryczkowski P (2018). Real-time full-field characterization of transient dissipative soliton dynamics in a mode-locked laser. Nat. Photon..

[16] Tikan A, Bielawski S, Szwaj C, Randoux S, Suret P (2018). Single-shot measurement of phase and amplitude by using a heterodyne time-lens system and ultrafast digital time-holography. Nat. Photon..

[17] Herr T (2014). Temporal solitons in optical microresonators. Nat. Photon..

[18] Yi X, Yang QF, Yang KY, Suh MG, Vahala K (2015). Soliton frequency comb at microwave rates in a high-Q silica microresonator. Optica.

[19] Brasch V (2016). Photonic chip-based optical frequency comb using soliton cherenkov radiation. Science.

[20] Wang PH (2016). Intracavity characterization of micro-comb generation in the single-soliton regime. Opt. Express.

[21] Joshi C (2016). Thermally controlled comb generation and soliton modelocking in microresonators. Opt. Lett..

[22] Obrzud E, Lecomte S, Herr T (2016). Temporal solitons in microresonators driven by optical pulses. Nat. Photon..

[23] Lobanov V (2016). Harmonization of chaos into a soliton in Kerr frequency combs. Opt. Express.

[24] Akhmediev, N. & Ankiewicz, A. *Dissipative solitons: from optics to biology and medicine*. (Springer, 2008).

[25] Okawachi Y (2012). Asynchronous single-shot characterization of high-repetition-rate ultrafast waveforms using a time-lens-based temporal magnifier. Opt. Lett..

[26] Lucas, E., Karpov, M., Guo, H., Gorodetsky, M. & Kippenberg, T. Breathing dissipative solitons in optical microresonators. *Nat. Commun*. **8**, 736 (2017). 10.1038/s41467-017-00719-w PMC5622060 28963496

[27] Diddams SA (2010). The evolving optical frequency comb. J. Opt. Soc. Am. B.

[28] Del’Haye P (2007). Optical frequency comb generation from a monolithic microresonator. Nature.

[29] Kippenberg TJ, Holzwarth R, Diddams S (2011). Microresonator-based optical frequency combs. Science.

[30] Suh MG, Yang QF, Yang KY, Yi X, Vahala KJ (2016). Microresonator soliton dual-comb spectroscopy. Science.

[31] Dutt A (2018). On-chip dual-comb source for spectroscopy. Sci. Adv..

[32] Marin-Palomo P (2017). Microresonator-based solitons for massively parallel coherent optical communications. Nature.

[33] Trocha P (2018). Ultrafast optical ranging using microresonator soliton frequency combs. Science.

[34] Suh MG, Vahala KJ (2018). Soliton microcomb range measurement. Science.

[35] Spencer DT (2018). An optical-frequency synthesizer using integrated photonics. Nature.

[36] Yang QF, Yi X, Yang KY, Vahala K (2017). Stokes solitons in optical microcavities. Nat. Phys..

[37] Wang Y (2017). Universal mechanism for the binding of temporal cavity solitons. Optica.

[38] Cole DC, Lamb ES, Delâ€™Haye P, Diddams SA, Papp SB (2017). Soliton crystals in Kerr resonators. Nat. Photon..

[39] Akhmediev N, Korneev V (1986). Modulation instability and periodic solutions of the nonlinear schrödinger equation. Theor. Math. Phys..

[40] Leo F, Gelens L, Emplit P, Haelterman M, Coen S (2013). Dynamics of one-dimensional Kerr cavity solitons. Opt. Express.

[41] Bao C (2016). Observation of fermi-pasta-ulam recurrence induced by breather solitons in an optical microresonator. Phys. Rev. Lett..

[42] Karpov M (2016). Raman self-frequency shift of dissipative Kerr solitons in an optical microresonator. Phys. Rev. Lett..

[43] Wang Y, Anderson M, Coen S, Murdoch SG, Erkintalo M (2018). Stimulated raman scattering imposes fundamental limits to the duration and bandwidth of temporal cavity solitons. Phys. Rev. Lett..

[44] Coddington I, Swann WC, Newbury NR (2009). Coherent linear optical sampling at 15 bits of resolution. Opt. Lett..

[45] Ferdous F, Leaird DE, Huang CB, Weiner A (2009). Dual-comb electric-field cross-correlation technique for optical arbitrary waveform characterization. Opt. Lett..

[46] Coddington I, Swann WC, Nenadovic L, Newbury NR (2009). Rapid and precise absolute distance measurements at long range. Nat. Photon..

[47] Coddington I, Newbury N, Swann W (2016). Dual-comb spectroscopy. Optica.

[48] Murata H, Morimoto A, Kobayashi T, Yamamoto S (2000). Optical pulse generation by electrooptic-modulation method and its application to integrated ultrashort pulse generators. IEEE J. Sel. Top. Quantum Electron..

[49] Durán V (2015). Ultrafast electrooptic dual-comb interferometry. Opt. Express.

[50] Lee H (2012). Chemically etched ultrahigh-Q wedge-resonator on a silicon chip. Nat. Photon..

[51] Kippenberg T, Spillane S, Vahala K (2004). Kerr-nonlinearity optical parametric oscillation in an ultrahigh-Q toroid microcavity. Phys. Rev. Lett..

[52] Yi X, Yang QF, Yang KY, Vahala K (2016). Theory and measurement of the soliton self-frequency shift and efficiency in optical microcavities. Opt. Lett..

[53] Yi X, Yang QF, Youl K, Vahala K (2016). Active capture and stabilization of temporal solitons in microresonators. Opt. Lett..

[54] Yi X (2017). Single-mode dispersive waves and soliton microcomb dynamics. Nat. Commun..

[55] Agrawal, G. P. *Nonlinear fiber optics* (Academic Press, 2007).

[56] Andrekson P (1991). Picosecond optical sampling using four-wave mixing in fibre. Electron. Lett..

[57] Lugiato LA, Lefever R (1987). Spatial dissipative structures in passive optical systems. Phys. Rev. Lett..

[58] Herr T (2014). Mode spectrum and temporal soliton formation in optical microresonators. Phys. Rev. Lett..

[59] Carlson, D. R. et al. An ultrafast electro-optic light source with sub-cycle precision. Preprint at http://arxiv.org/abs/1711.08429 (2017).

[60] Lucas, E. et al. Spatial multiplexing of soliton microcombs. Preprint at http://arxiv.org/abs/1804.03706 (2018).

[61] Cai M, Painter O, Vahala KJ (2000). Observation of critical coupling in a fiber taper to a silica-microsphere whispering-gallery mode system. Phys. Rev. Lett..

[62] Spillane SM, Kippenberg TJ, Painter OJ, Vahala KJ (2003). Ideality in a fiber-taper-coupled microresonator system for application to cavity quantum electrodynamics. Phys. Rev. Lett..

