# Supplementary material for: Imaging soliton dynamics in optical microcavities
Source: Nat Commun. 2018 Sep 3;9:3565. doi: 10.1038/s41467-018-06031-5 (PMC6120930; doi:10.1038/s41467-018-06031-5)
Supplement: Supplementary file 1 — Description of Additional Supplementary Files [file 41467_2018_6031_MOESM1_ESM.pdf]

Description of Additional Supplementary Files

File Name: Supplementary Movie 1
Description: Movie showing soliton motion in figure 1d.
